# PAGln, an Atrial Fibrillation-Linked Gut Microbial Metabolite, Acts as a Promoter of Atrial Myocyte Injury

**DOI:** 10.3390/biom12081120

**Published:** 2022-08-15

**Authors:** Chen Fang, Kun Zuo, Kaicheng Jiao, Xiaoming Zhu, Yuan Fu, Jiuchang Zhong, Li Xu, Xinchun Yang

**Affiliations:** Heart Center & Beijing Key Laboratory of Hypertension, Beijing Chaoyang Hospital, Capital Medical University, Beijing 100020, China

**Keywords:** atrial fibrillation, phenylacetylglutamine, atrial myocytes, apoptosis, ROS

## Abstract

Phenylacetylglutamine (PAGln), a gut microbiota (GM)-derived metabolite, is associated with cardiovascular disease. Studies have shown that disordered GM participated in the progression of atrial fibrillation (AF), but the relationship between PAGln and AF is unclear. This study investigated the characteristics of PAGln in AF patients and its impact on atrial myocytes. Based on our previous metagenomic data, the relative abundance of *porA*, a critical bacterial enzyme for PAGln synthesis, exhibited an increased tendency in AF. In an independent cohort consisting of 42 controls without AF and 92 AF patients, plasma PAGln levels were higher in AF patients than in controls (*p* < 0.001) by immunoassay. Notably, PAGln exerted a predictive potential of AF with an AUC of 0.774 (*p* < 0.001), and a predictive model constructed based on the PAGln and Taiwan AF score further improved the predictive potential. Furthermore, a positive correlation was determined between PAGln and LA diameter. Subsequently, the effect of PAGln intervention was examined on HL-1 cells in vitro, revealing that PAGln increased apoptosis, reactive oxygen species (ROS) production, CaMKII and RyR2 activation and decreased cell viability. In conclusion, increased PAGln was associated with AF, and PAGln might contribute to the AF pathogenesis by promoting oxidative stress and apoptosis in atrial myocytes.

## 1. Introduction

Atrial fibrillation (AF), the most common cardiac arrhythmia, affects more than 33 million patients over the age of 55, with a lifetime incidence of 37% [1]. Although great progress has been made in interventions for cardioversion and catheter ablation, the recurrence rate of AF remains high [1]. Further exploration of AF novel risk factors and complementary therapeutic targets is required.

Recently, gut microbiota (GM)–heart crosstalk has attracted significant attention to cardiovascular pathologies [2]. GM as an emerging virtual endocrine organ is involved in host homeostasis [3]. Aberrant GM structure and function are associated with cardiovascular disease (CVD), including AF, hypertension (HTN), heart failure (HF) and coronary artery disease (CAD) [4,5,6]. In particular, metabolites derived from GM, such as trimethylamine-N-oxide (TMAO), short-chain fatty acids (SCFAs), bile acids and lipopolysaccharide (LPS), exert pivotal regulations in the disordered GM–host interaction [7,8].

Another GM-dependent metabolite, phenylacetylglutamine (PAGln), has been determined to predict incident CVD risks in large-scale clinical cohorts, which fosters CVD-relevant phenotypes via α2A, α2B and β2 adrenergic receptors (β2ARs) [3,8,9]. Notably, these adrenergic receptors are expressed in the human atrium, and the stimulation leads to the initiation and maintenance of AF [10,11,12]. For example, selective activation of β2AR induces various types of cardiac tachyarrhythmias, including the spontaneous onset of AF [13,14,15,16,17]. Therefore, PAGln may be involved in AF development and might even be a promising target for AF therapy.

Our previous research revealed imbalanced gut microbial and metabolic patterns in AF patients, but whether GM-derived PAGln acts on AF progression is unknown [18]. In the present study, we analyzed the alteration in the relative abundance of *porA*, a critical gut microbial synthesis enzyme of PAGln [9], based on our previously published AF metagenomic data. Then, the plasma PAGln level was measured by immunoassay. Subsequently, the direct effect of PAGln on atrial myocytes was observed, including CaMKII and RyR2 activities, apoptosis, ROS generation and cell viability.

## 2. Materials and Methods

### 2.1. Relative Abundance of Gut Microbial PorA Enzyme

The functional annotation and relative abundance of *porA* enzyme in 100 fecal samples (50 controls without AF and 50 AF patients) were obtained from our previously published metagenomic data [18].

### 2.2. Study Population

A total of 134 participants with or without AF were recruited in Beijing Chaoyang Hospital, including 42 controls without AF and 92 AF patients. The diagnosis of AF was based on 2020 ESC guidelines for diagnosing and managing AF [19]. The exclusion criteria were as follows: valvular heart disease, severe infection, thyroid diseases, acute coronary syndrome, autoimmune disease, renal failure, hepatic failure and tumors. The study conformed to the principles of the Declaration of Helsinki and was approved by the Ethics Committee of Beijing Chaoyang Hospital affiliated to Capital Medical University. All participants provided informed consent.

### 2.3. Clinical Characteristics

Baseline clinical data were collected from all participants at enrollment, including age, gender, body mass index (BMI), smoking history, drinking history and medical history. Echocardiography, hepatic and renal function indexes and blood lipid levels were recorded.

### 2.4. Plasma PAGln Measurement

Blood samples were obtained from antecubital veins in the early morning using EDTA anticoagulation vacuum tubes. Plasma samples were collected after centrifugation at 4 °C, 3000 rpm for 10 min, and then immediately stored at −80 °C in 1.5 mL microcentrifuge tubes until analysis. Plasma PAGln levels were measured using an enzyme-linked immunosorbent assay kit (MLBio, Shanghai, China), following the manufacturer’s protocol.

### 2.5. Cell Culture and Intervention

Mouse atrial myocytes HL-1 (BNCC, Henan, China) were cultured in Dulbecco’s modified eagle medium (DMEM) (Hyclone, SH30022.01), containing 10% fetal bovine serum (FBS) and 1% penicillin and streptomycin (P/S) in a humidified incubator at 37 °C under 5% CO_2_ [20,21]. Subsequent experiments were performed after HL-1 cells were stimulated with or without PAGln (MedChemExpress, Monmouth Junction, NJ, USA) for 24 h.

### 2.6. Apoptosis Assays

The Annexin V-FITC/PI Apoptosis Detection Kit was used to detect the apoptosis rate of HL-1 cells according to the manufacturer’s instructions. Following treatment for 24 h, the cells were collected, washed with PBS and resuspended in Annexin V-FITC/PI binding buffer. After 15 min incubation at room temperature in the dark, HL-1 cells were analyzed by Novocyte flow cytometer. All experiments were repeated three times.

### 2.7. Reactive Oxygen Species Assay

The generation of cellular reactive oxygen species (ROS) was measured by Reactive Oxygen Species Assay Kit. Cells were harvested by trypsin and washed with pre-cold PBS, then resuspended and co-incubated with serum-free cultured medium containing 10 μM 2,7-Dichlorodi -hydrofluorescein diacetate (DCFH-DA) at 37 °C for 20 min in the dark. Cells were washed twice with the serum-free medium post incubation. After resuspending in PBS, cells were immediately detected using Novocyte flow cytometer.

### 2.8. MTT Assay

Cell viability was detected by MTT assay. HL-1 cells were cultured and treated for 24 h in 96-well plates and incubated with 10 μL of MTT for 4 h. Then, 100 μL of Formazan lysis solution was added, and they continued to be incubated until all the purple crystals dissolved. The absorbance was measured at 570 nm with a microplate reader (ELx808, BioTek, Winooski, VT, USA).

### 2.9. Measurement of Superoxide Dismutase (SOD) and NADPH Oxidase (NOX) Activities

Cellular protein was extracted after lysis and centrifugation, and the BCA kit (Thermo Scientific, Waltham, MA, USA) was used to determine protein concentrations. SOD and NOX activities were measured by SOD (Nanjing Jiancheng Bioengineering Institute, Nanjing, China, A001-3) and NOX detection assay kits (Solarbio, Beijing, China, BC0630) according to the manufacturer’s introductions.

### 2.10. Western Blot Analysis

Cellular proteins were separated by sodium dodecyl sulfate–polyacrylamide gel electrophoresis (SDS-PAGE) and transferred to nitrocellulose membranes. After blocking with 5% skim milk for 1 h at room temperature, the membranes were incubated overnight at 4 °C with the primary antibodies against p-CaMKII, CaMKII, p-RyR2, RyR2 and GAPDH. Subsequently, membranes were incubated with secondary antibodies for 1 h, detected using the Odyssey infrared imaging system (LI-COR, Lincoln, NE, USA) and analyzed by ImageJ software. GAPDH was used as an endogenous control. Antibodies were obtained from Cell Signaling Technology, Proteintech, and ABclonal.

### 2.11. Statistical Analysis

Continuous variables were expressed as mean  ±  standard deviation (SD) or median (quartile). Student’s *t*-test and Mann–Whitney test were performed for continuous variables with normal or nonnormal distribution, respectively. Categorical variables were presented as numbers (percentages) and analyzed using chi-square test. Correlation was evaluated using Pearson and Spearman correlation analysis. Receiver operating characteristic (ROC) curves were constructed and the area under curve (AUC) was used to evaluate the association between plasma PAGln and AF. Multivariate logistic regression based on variables selected by univariate analysis (*p* < 0.1) was used to identify risk factors for AF. Logistic regression analysis was performed to construct the combined predictive model. Net reclassification index (NRI) and integrated discrimination (IDI) were calculated to compare the predictive values among these models. All statistical analyses were performed using SPSS version 25.0 (IBM Corporation, Armonk, NY, USA), R software (version 3.6.3) and MedCalc (V19.6.4). A *p*-value < 0.05 (two-sided) was considered statistically significant.

## 3. Results

### 3.1. Association between PAGln and AF

**Figure 1:**
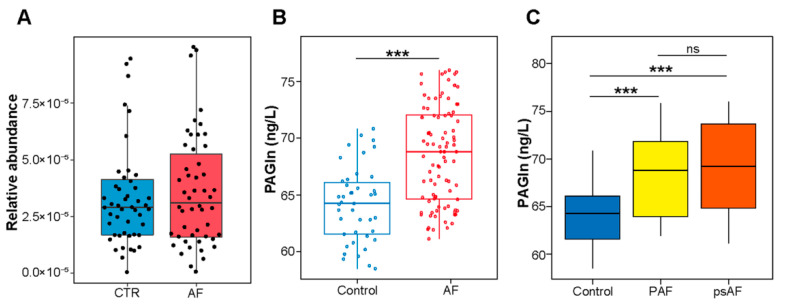
PAGln was significantly associated with AF. (**A**) Increased relative abundance of gut microbial *porA* enzyme in AF fecal samples. *p* = 0.493, Student’s *t*-test. (**B**) Higher plasma PAGln levels in AF patients than controls without AF. (**C**) Levels of plasma PAGln in PAF, psAF and control groups. PAF, paroxysmal atrial fibrillation; psAF, persistent atrial fibrillation. *** *p* < 0.001; ns, no significance.

Based on our previous AF metagenomic data [18], the relative abundance of *porA*, a critical PAGln-synthesis-related GM enzyme [9], was assessed. *porA* exhibited higher tendency in AF compared with controls (*p* = 0.493) (Figure 1A).

To further determine the characteristic of PAGln in AF, plasma PAGln levels were measured. The levels of plasma PAGln were significantly higher in the AF patients compared to the controls (68.60 ± 4.31 vs. 64.18 ± 3.30 ng/L, *p* < 0.001) (Figure 1B). Plasma PAGln was significantly correlated to AF onset (R = 0.440, *p* < 0.001, Spearman’s correlation). Based on the duration of the episodes, AF patients were divided into paroxysmal (PAF; lasting < 7 days) and persistent (psAF; lasting > 7 days) AF groups [19]. There was also an upward trend in plasma PAGln level in paroxysmal (PAF) and persistent AF (psAF) groups (PAF vs. psAF group, 68.55 (63.54, 71.87) vs. 69.23 (64.79, 73.58) ng/L, *p* = 0.269), with a significant difference compared to the control group (*p* < 0.001) (Figure 1C). Detailed comparisons of baseline characteristics between control, AF, PAF and psAF groups were shown in Table 1 and Table S1.

Based on the univariate logistic analysis, the variables of male, DM, HGB, age, AST, sCr and PAGln (*p* < 0.1) were selected for multivariate logistic analysis indicating that PAGln plasma levels (odds ratio (OR): 1.437, 95% confidence interval (CI): 1.143–1.806, *p* = 0.002) and age were independent risk factors for AF (OR: 1.103, 95% CI: 1.026–1.186, *p* = 0.008) (Table 2). These results suggest that GM-derived PAGln was closely related to AF occurrence.

### 3.2. Plasma PAGln in the Prediction of AF

**Figure 2:**
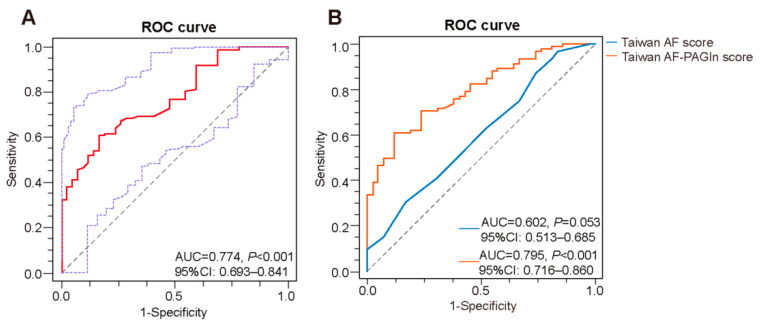
Predictive potential of PAGln for AF. (**A**) ROC analysis on plasma PAGln in predicting presence of AF (AUC = 0.774, 95% CI: 0.693–0.841, *p* < 0.001). (**B**) ROC curve of the Taiwan AF score (blue) (AUC = 0.602, 95% CI: 0.513–0.685; *p* = 0.053) and Taiwan AF-PAGln score (orange) (AUC = 0.795, 95% CI: 0.716–0.860, *p* < 0.001).

ROC curve analysis revealed that plasma PAGln exerted a predictive potential of AF with AUC of 0.774 (95%CI: 0.693–0.841, *p* < 0.001, cut-off value: 66.86 ng/L, sensitivity: 60.87%, specificity: 83.33%) (Figure 2A). Meanwhile, the Taiwan AF score, which included age, male gender, HTN, heart failure (HF), CAD, end-stage renal disease and alcoholism, has been recently reported as a clinical prediction model for the incident AF, especially for Asian patients [22]. In this study, the area under the receiver operating characteristic curve (AUROC) of the Taiwan AF score in AF prediction was 0.602 (95% CI: 0.513–0.685, *p* = 0.053). To assess whether PAGln could improve the clinical prediction model, a novel combined model based on PAGln and Taiwan AF score, named Taiwan AF-PAGln score, was constructed via logistic regression analysis as follows: Taiwan AF-PAGln score = (−18.573 × (Intercept)) + (0.283 × (PAGln)) + (0.167 × (Taiwan AF score)) with the AUROC of 0.795 (95% CI: 0.716–0.860, *p* < 0.001) (Figure 2B). Compared to the Taiwan AF score and PAGln alone, the Taiwan AF-PAGln score improved AF prediction (Taiwan AF-PAGln score vs. Taiwan AF score, AUROC: 0.795 vs. 0.602, *p* < 0.001, NRI: 75.88%, *p* < 0.001, IDI: 19.59%, *p* < 0.001; Taiwan AF-PAGln score vs. PAGln, AUROC: 0.795 vs. 0.774, *p* = 0.223, NRI: 21.33%, *p* = 0.247, IDI: 2.32%, *p* = 0.127). Thus, plasma PAGln can theoretically improve the performance of the Taiwan AF model.

### 3.3. Correlation between Plasma PAGln and Structural Parameters of the Left Atrium

**Figure 3:**
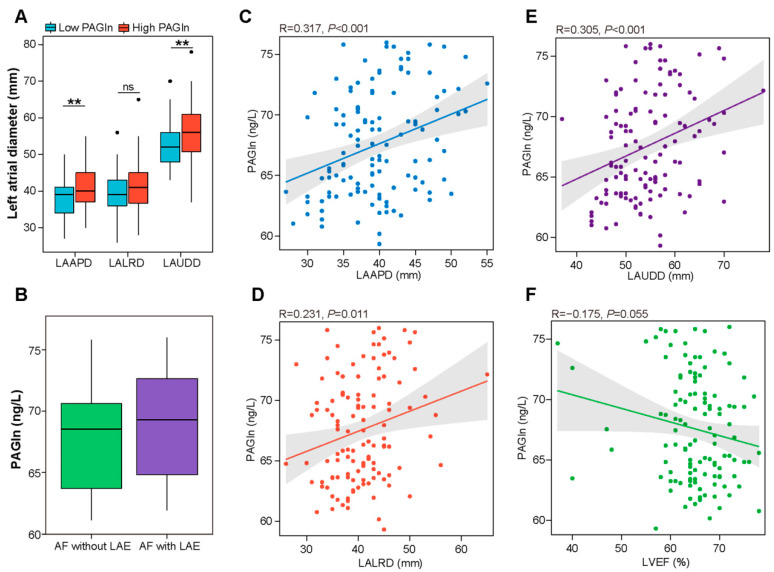
Correlations between left atrial dimension, LVEF and plasma PAGln. (**A**) Higher left atrial diameters (LAAPD, LALRD and LAUDD) in the high-PAGln group (>66.86 ng/L) than in the low-PAGln group (≤66.86 ng/L). LAAPD, left atrial anteroposterior diameter, *p* = 0.002; LAUDD, left atrial up and down diameter, *p* = 0.002; LALRD, left atrial left and right diameter, *p* = 0.056. (**B**) Plasma levels of PAGln were elevated in AF patients with LAE compared to the levels in AF patients without LAE. *p* = 0.337. (**C**–**E**) Plasma PAGln was positively associated with LAAPD (R = 0.317, *p* < 0.001), LALRD (R = 0.231, *p* = 0.011) and LAUDD (R = 0.305, *p* < 0.001) based on Pearson correlation analysis. (**F**) Plasma PAGln was negatively related to LVEF (R = −0.175, *p* = 0.055). Pearson correlation analysis. **, *p* < 0.01; ns, no significance.

To explore the potential role of PAGln in AF progression, we assessed the association between plasma PAGln levels and left atrial structural remodeling, the pathophysiological basis of AF [23]. In the present study, 29 controls and 92 patients in the AF group underwent transthoracic echocardiography. According to the above optimal cut-off value, participants were divided into low- (≤66.86 ng/L) and high- (>66.86 ng/L) PAGln groups. As shown in Figure 3A, the high-PAGln group had significantly elevated left atrial diameters, including left atrial anteroposterior diameter (LAAPD) (41.33 ± 5.63 vs. 38.21 ± 5.38 mm, *p* = 0.002), left atrial up and down diameter (LAUDD) (56.40 ± 7.59 vs. 52.54 ± 6.02 mm, *p* = 0.002) and left atrial left and right diameter (LALRD) (41.77 ± 6.75 vs. 39.66 ± 5.20 mm, *p* = 0.056) compared to the low-PAGln group. Furthermore, plasma PAGln levels were positively correlated with LAAPD (R = 0.317, *p* < 0.001) (Figure 3C), LALRD (R = 0.231, *p* = 0.011) (Figure 3D) and LAUDD (R = 0.305, *p* < 0.001) (Figure 3E). Moreover, there was a negative correlation between plasma PAGln levels and LVEF (R = −0.175, *p* = 0.055) (Figure 3F). AF patients with a left atrial enlargement (LAE) had higher plasma PAGln levels compared to AF patients without LAE (68.87 ± 4.39 vs. 67.91 ± 4.10 ng/L, *p* = 0.337) (Figure 3B). Although the difference was not statistically significant, the results might suggest that PAGln may be involved in AF-related atrial remodeling.

### 3.4. PAGln Aggravates Atrial Myocyte Oxidative Stress and Apoptosis

**Figure 4:**
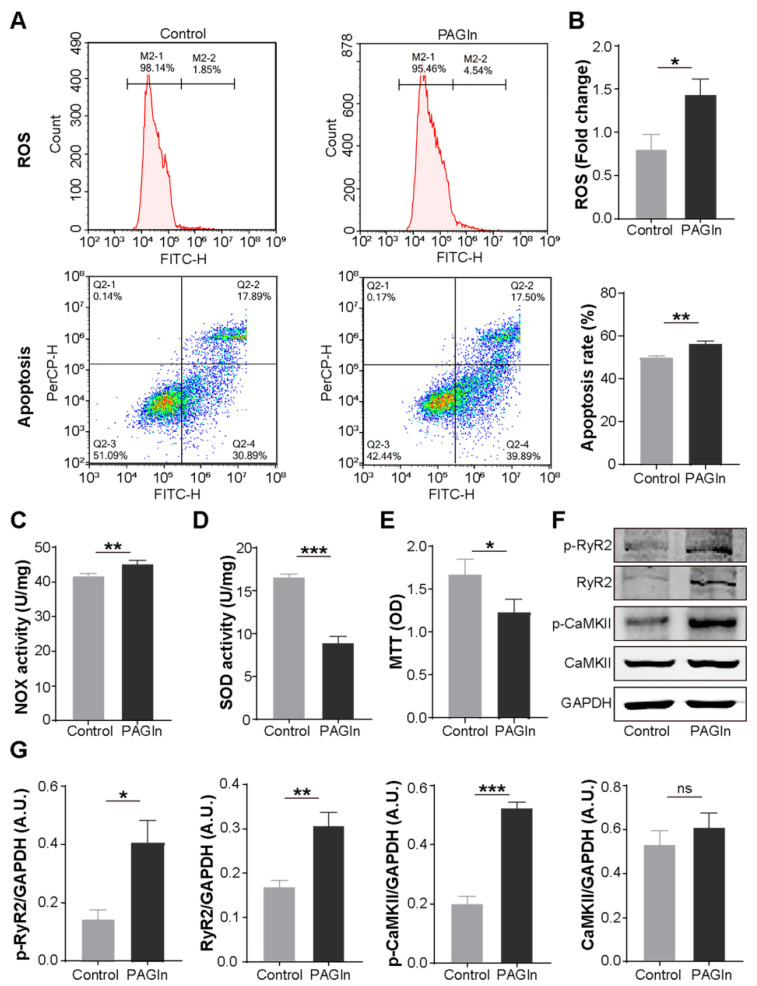
The levels of apoptosis, ROS production, CaMKII and RyR2 activation and cell viability were detected in mouse atrial myocytes treated with or without PAGln. (**A**) Representative flow cytometry results of apoptosis and ROS levels in control and PAGln groups. (**B**) PAGln treatment enhanced ROS production and apoptosis. *n* = 3. (**C**) Increased NOX activity in HL-1 cells treated with PAGln. *n* = 4. (**D**,**E**) PAGln treatment decreased SOD activity and cell viability. *n* = 4. (**F**,**G**) Representative Western blot images and the expression levels of p-CaMKII, CaMKII, p-RyR2 and RyR2 in HL-1 cells treated with or without PAGln. GAPDH was used as an endogenous control. *n* = 4–8; *, *p* < 0.05; **, *p* < 0.01; ***, *p* < 0.001; ns, no significance.

Next, we evaluated the effect of PAGln on atrial myocytes. Compared with the control group, PAGln (100 μM, 24 h) intervention significantly increased ROS generation (1.44 ± 0.18 vs. 0.80 ± 0.17 fold change; *p* = 0.011), NOX activity, a major source of excess ROS in the cardiovascular system [24], and apoptosis (56.25 ± 1.18 vs. 49.76 ± 0.93%, *p* = 0.002) in mouse HL-1 cells (Figure 4A–C). Moreover, treating HL-1 cells with PAGln for 24 h induced a striking decrease in SOD activity, a myocardial endogenous antioxidant [24], and the MTT values (Figure 4D,E), showing that PAGln could dramatically impair atrial myocyte viability. Therefore, PAGln could directly damage atrial myocytes.

### 3.5. PAGln Induces Atrial Myocyte Activation of CaMKII and RyR2

The critical roles of CaMKII and RyR2 activation and phosphorylation in AF progression have been demonstrated [25]. In the present study, we found that PAGln treatment (100 μM, 24 h) significantly induced the expression of p-CaMKII, p-RyR2 and RyR2 in HL-1 cells, while the level of CaMKII increased in the PAGln group without statistical significance (Figure 4F,G).

## 4. Discussion

The current study provided preliminary support for the role of GM-derived PAGln in AF development, revealing that AF patients had elevated relative abundance of *porA* enzyme, a key PAGln-synthesis-related GM enzyme, in feces and significantly increased PAGln levels in circulation. As an independent risk factor of AF, plasma PAGln was positively associated with left atrial enlargement including LAAPD, LAUDD and LALRD while negatively related to LVEF. AF patients with LAE exerted higher plasma PAGln levels than those without LAE. Furthermore, PAGln induced ROS generation, CaMKII and RyR2 activation, apoptosis and impaired cell viability in atrial myocytes. PAGln may be a potential predictive marker and promising therapeutic target for AF.

Altered gut microbial composition and metabolic patterns are involved in AF development and recurrence [18,26,27,28]. GM-derived metabolite PAGln has been found to be substantially elevated in end-stage renal disease, acute ischemic stroke and coronary artery disease (CAD) with stent stenosis and related to CVD and incident major adverse cardiovascular events including myocardial infarction, stroke and death [9,29,30]. Likewise, our data also confirmed the association between AF and PAGln. AF patients had increased relative abundances of fecal *porA*, a critical bacterial enzyme for PAGln synthesis. Plasma PAGln levels were significantly elevated in AF patients and exerted a predictive potential for AF. The newly constructed Taiwan AF-PAGln score could better identify patients at high risk of AF, and its clinical value needs to be further confirmed in the future. Furthermore, this study in vitro demonstrated that PAGln intervention induced ROS generation concomitant increased NOX and decreased SOD activity, CaMKII and RyR2 activation and apoptosis in mouse atrial myocytes, providing direct evidence for the adverse effects of PAGln in AF.

Increasing evidence indicates ROS, a product of oxidative stress, has a vital role in AF progression and atrial remodeling [31]. ROS accumulation could promote the activation of the enzyme calmodulin kinase II (CaMKII) and RyR2, Ca^2+^ release and calcium overload [31]. Enhanced CaMKII and RyR2 activation and calcium overload induce a proarrhythmic circumstance by favoring cell membrane hyperexcitability and afterdepolarizations [32,33,34,35]. Cardiomyocyte apoptosis has been documented with AF development by inducing atrial remodeling and reducing electrical conduction velocity [36]. Additionally, oxidative stress, together with myocardial apoptosis, promoting atrial fibrosis and inflammation, serves as an essential driver of atrial structural remodeling to create a substrate for AF [36,37,38,39,40]. Any persistent change in atrial structure and function constitutes atrial remodeling, promoting the occurrence or maintenance of AF [41]. Although PAGln-exacerbated apoptosis and ROS production were confirmed in mouse-derived atrial myocytes, these results also provide preliminary evidence for the adverse effects of PAGln on human AF development. Meanwhile, LAE represents maladaptive structural and functional alteration and is characteristic of atrial remodeling, which in turn promotes AF progression and serious decline in left ventricular function [23,42]. Consistently, our data revealed that plasma PAGln levels were positively linked to indexes (LAAPD, LAUDD and LALRD) related to LA size and negatively related to LVEF. AF patients with LAE had higher plasma PAGln than AF patients without LAE. Thus, PAGln is involved in AF occurrence and atrial remodeling and may be a promising therapeutic target for AF; however, the specific mechanism remains unclear.

Recently, PAGln was reported to enhance platelet responsiveness and thrombosis potential in whole blood, isolated platelets and animal models of arterial injury via α2A, α2B and β2 ARs [9]. Activation of β2 AR, which has been confirmed to be located in the atrium, potentiates spontaneous calcium release and is linked to atrial arrhythmias [13,43]. Thus, β2 AR may be one of the signals mediating the PAGln effect on AF, which needs to be further confirmed.

There are some limitations to this study. This study was a cross-sectional study with a limited sample size and a lack of mechanism exploration. Further large-scale prospective cohort studies and mechanistic research are warranted.

## 5. Conclusions

Plasma PAGln levels were significantly associated with AF and LAE. PAGln might be involved in AF progression through promoting atrial myocyte apoptosis and ROS generation. PAGln exerted a predictive value and may be a promising therapeutic target for AF.

## Figures and Tables

**Table 1:** Baseline clinical characteristics of the study participants with and without AF.

	Control	AF	*p* Value
Number	42	92	
Male, %	15 (35.71)	50 (54.35)	0.062
HTN, %	24 (57.14)	49 (53.26)	0.712
DM, %	5 (11.90)	25 (27.17)	0.073
CAD, %	0 (0.00)	7 (7.61)	0.098
Smoking, %	9 (21.43)	15 (16.30)	0.476
Drinking, %	10 (23.81)	14 (15.22)	0.235
Age, years	61.52 ± 9.83	65.00 ± 9.88	0.061
BMI, kg/m^2^	25.99 ± 3.40	26.07 ± 3.60	0.918
WBC, ×10^9^/L	5.90 ± 1.17	5.95 ± 1.49	0.883
HGB, g/L	129.28 ± 9.44	138.87 ± 17.74	0.002 **
PLT, ×10^9^/L	224.56 ± 40.10	204.10 ± 60.54	0.173
TC, mmol/L	4.36 ± 1.06	4.06 ± 0.88	0.101
TG, mmol/L	0.95 (0.77, 1.89)	1.19 (0.92, 1.49)	0.257
AST, U/L	17.40 ± 6.67	19.86 ± 6.29	0.053
ALT, U/L	13.90 (10.40, 19.30)	17.00 (13.00, 23.00)	0.015 *
sCr, μmol/L	65.39 ± 15.41	70.57 ± 14.49	0.076
cTNI, ng/mL	0.00 (0.00, 0.00)	0.00 (0.00,0.01)	0.266

Data are presented as number (%), mean ± SD and median (quartile). ALT, alanine aminotransfease; AST, aspartate aminotransferase; BMI, body mass index; CAD, coronary artery disease; cTNI, cardiac troponin I; DM, diabetes mellitus; HGB, hemoglobin; HTN, hypertension; PLT, platelet; sCr, serum creatinine; TC, total cholesterol; TG, triglyceride; WBC: white blood cell. *, *p* < 0.05; **, *p* < 0.01.

**Table 2:** Association between clinical variables and AF.

	Univariate Analysis		Multivariate Analysis	
	OR (95% CI)	*p* Value	OR (95% CI)	*p* Value
Male	0.467 (0.220–0.991)	0.047 *		
DM	0.362 (0.128–1.025)	0.056		
Age	1.036 (0.998–1.075)	0.063	1.103 (1.026–1.186)	0.008 **
HGB	1.037 (1.003–1.072)	0.032 *		
AST	1.081 (0.999–1.170)	0.054		
sCr	1.026 (0.997–1.055)	0.079		
PAGln	1.331 (1.183–1.502)	<0.001 ***	1.437 (1.143–1.806)	0.002 **

AST, aspartate aminotransferase; DM, diabetes mellitus; HGB, hemoglobin; PAGln, phenylacetylglutamine. *, *p* < 0.05; **, *p* < 0.01; ***, *p* < 0.001.

## Data Availability

The data that support the findings of this study are available from the corresponding author upon reasonable request.

## Supplementary Materials

The following supporting information can be downloaded at: https://www.mdpi.com/article/10.3390/biom12081120/s1, Table S1: Baseline characteristics of patients with paroxysmal and persistent AF.
