# Supplementary material for: PAGln, an Atrial Fibrillation-Linked Gut Microbial Metabolite, Acts as a Promoter of Atrial Myocyte Injury
Source: Biomolecules. 2022 Aug 15;12(8):1120. doi: 10.3390/biom12081120 (PMC9405855; doi:10.3390/biom12081120)
Supplement: Supplementary file 1 [file biomolecules-12-01120-s001.zip › biomolecules-1812182-supplementary.pdf]

Table S1

Baseline characteristics of patients with paroxysmal and persistent AF.

	Paroxysmal AF	Persistent AF	*P* value
Number	46	46	
Male, %	24 (52.17)	26 (56.52)	0.834
HTN, %	20 (43.48)	29 (63.04)	0.094
DM, %	12 (26.09)	13 (28.26)	1.000
CAD, %	3 (6.52)	4 (8.70)	1.000
Smoking, %	7 (15.22)	8 (17.39)	1.000
Drinking, %	5 (10.87)	9 (19.57)	0.385
Age, years	65.41 ± 10.68	64.59 ± 9.12	0.691
BMI, kg/m^2	25.42 ± 3.69	26.70 ± 3.43	0.105
WBC, × 10^9/L	5.78 ± 1.46	6.12 ± 1.52	0.270
HGB, g/L	136.11 ± 16.36	141.63 ± 18.78	0.136
PLT, × 10^9/L	206.02 ± 52.07	202.17 ± 68.51	0.762
TC, mmol/L	3.97 ± 0.86	4.14 ± 0.91	0.368
TG, mmol/L	1.20 (0.84, 1.48)	1.19 (0.93, 1.69)	0.555
AST, U/L	20.00 (15.75, 23.00)	18.00 (15.75, 20.25)	0.194
ALT, U/L	17.00 (12.00, 24.25)	17.00 (14.00, 22.00)	0.959
sCr, µmol/L	67.25 ± 14.89	73.90 ± 13.43	0.027
cTNI, ng/mL	0.00 (0.00, 0.00)	0.00 (0.00, 0.01)	0.450

Data are presented as number (%), mean ± SD and median (quartile). ALT, alanine aminotransfease; AST, aspartate aminotransferase; BMI, body mass index; CAD, coronary artery disease, cTNI, cardiac troponin I; DM, diabetes mellitus; HGB, hemoglobin; PLT, platelet; sCr, serum creatinine; TC, total cholesterol; TG, triglyceride; WBC: white blood cell.

Original western blots of Figure 4F

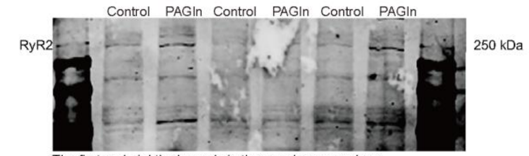

The first and eighth channels in the panel were markers.
The sixth and seventh channels were immunoblots of RyR2
in the manuscript.

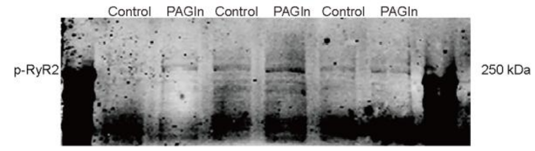

The first and eighth channels in the panel were markers.
The fourth and fifth channels were immunoblots of p-RyR2
in the manuscript.

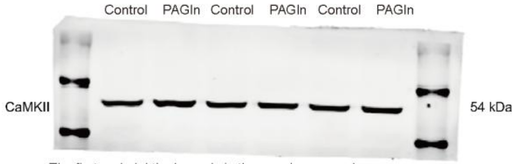

The first and eighth channels in the panel were markers.
The second and third channels were immunoblots of CaMKII
in the manuscript.

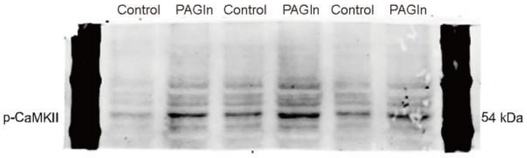

The first and eighth channels in the panel were markers.
The fourth and fifth channels were immunoblots of p-CaMKII
in the manuscript.

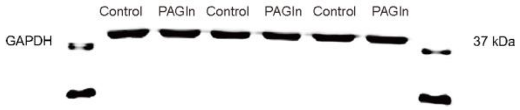

The first and eighth channels in the panel were markers.
The second and third channels were immunoblots of GAPDH
in the manuscript.
